# Concomitant pancreatic endocrine neoplasm and intraductal papillary mucinous neoplasm: a case report and literature review

**DOI:** 10.1186/1477-7819-11-75

**Published:** 2013-03-21

**Authors:** Yoshie Kadota, Masahiro Shinoda, Minoru Tanabe, Hanako Tsujikawa, Akihisa Ueno, Yohei Masugi, Go Oshima, Ryo Nishiyama, Masayuki Tanaka, Kisho Mihara, Yuta Abe, Hiroshi Yagi, Minoru Kitago, Osamu Itano, Shigeyuki Kawachi, Koichi Aiura, Akihiro Tanimoto, Michiie Sakamaoto, Yuko Kitagawa

**Affiliations:** 1 Department of Surgery, School of Medicine, 35 Shinanomachi, Shinjuku, Tokyo 160-8582, Japan; 2 Department of Pathology, School of Medicine, 35 Shinanomachi, Shinjuku, Tokyo, 160-8582, Japan; 3 Department of Diagnostic Radiology Keio University, School of Medicine, 35 Shinanomachi, Shinjuku, Tokyo, 160-8582, Japan; 4 Department of Surgery, Kawasaki Municipal Hospital, 12-1 Shinkawadori, Kawasaki-shi, Kawasaki-ku, Kanagawa-ken, 210-0013, Japan

**Keywords:** Pancreatic endocrine neoplasm, Intraductal papillary mucinous neoplasm

## Abstract

We report a case of concomitant pancreatic endocrine neoplasm (PEN) and intraductal papillary mucinous neoplasm (IPMN). A 74-year-old man had been followed-up for mixed-type IPMN for 10 years. Recent magnetic resonance images revealed an increase in size of the branch duct IPMN in the pancreas head, while the dilation of the main pancreatic duct showed minimal change. Although contrast-enhanced computed tomography and magnetic resonance imaging did not reveal any nodules in the branch duct IPMN, endoscopic ultrasound indicated a suspected nodule in the IPMN. A malignancy in the branch duct IPMN was suspected and we performed pylorus-preserving pancreatoduodenectomy with lymphadenectomy. The resected specimen contained a cystic lesion, 10 x 10 mm in diameter, in the head of the pancreas. Histological examination revealed that the dilated main pancreatic duct and the branch ducts were composed of intraductal papillary mucinous adenoma with mild atypia. No evidence of carcinoma was detected in the specimen. Incidentally, a 3-mm nodule consisting of small neuroendocrine cells was found in the main pancreatic duct. The cells demonstrated positive staining for chromogranin A, synaptophysin, and glucagon but negative staining for insulin and somatostatin. Therefore, the 3-mm nodule was diagnosed as a PEN. Since the mitotic count per 10 high-power fields was less than 2 and the Ki-67 index was less than 2%, the PEN was pathologically classified as low-grade (G1) according to the 2010 World Health Organization (WHO) criteria. Herein, we review the case and relevant studies in the literature and discuss issues related to the synchronous occurrence of the relatively rare tumors, PEN and IPMN.

## Background

Pancreatic endocrine neoplasm (PEN) and intraductal papillary neoplasm (IPMN) are both relatively rare tumors among the primary pancreatic neoplasms, with reported frequencies of approximately 0.4 per 100,000 and 1 per 100,000, respectively [1,2]. There have been only a few published reports of cases in which PEN and IPMN were present concomitantly [3-10]. We encountered a rare case who underwent pancreatoduodenectomy for pancreas head IPMN and whose postoperative pathological examination revealed the existence of concomitant PEN. We present this case and discuss issues related to the prevalence and tumorigenesis based on a review of published literature.

## Case presentation

**Figure 1:**
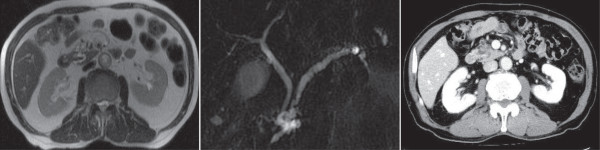
**Preoperative imaging diagnoses. A**) Magnetic resonance imaging findings (axial, T2-weighted image). The image indicated by a rectangle is illustrated as an inset in the lower right. A black curved line in the illustration indicates the main pancreas duct (MPD). A cystic lesion, illustrated as a cluster of gray areas, is seen around the main pancreas duct. This lesion is a cluster of multiple dilated branch pancreas ducts (BPD) and has been diagnosed as branch intraductal papillary mucinous neoplasm. **B**) Magnetic resonance cholangiopancreatography findings. The image is illustrated as an inset in the lower right. Black lines in the illustration indicate the main pancreas duct (MPD), the biliary tree and the gallbladder. The main pancreatic duct is dilated (7 mm) and also is markedly curved like an S-shape in the pancreas head. A lesion consisting of multiple dilated branch pancreas ducts (BPD), illustrated as a cluster of gray areas, is seen in the pancreas head. **C**) Contrast-enhanced computed tomography findings in the portal venous phase. A markedly curved main pancreas duct and multiple dilated branch pancreas ducts (indicated by arrows) are seen in the pancreas head. No enhanced nodules are seen in either the main pancreas duct or dilated branch pancreas ducts.

A 74-year-old man was admitted to our hospital for the examination of IPMN in the pancreas head. A cystic lesion, 6 mm in diameter, in the pancreas head was initially revealed when he underwent ultrasonography for a routine checkup for fatty liver 10 years prior. Magnetic resonance imaging revealed the cystic lesion and a slightly dilated main pancreas duct (6 mm). The patient was diagnosed with mixed-type (both main and branch pancreatic ducts involved) IPMN and has been carefully followed-up by magnetic resonance imaging every six months for the last 10 years. The most recent magnetic resonance images revealed an increase, relative to the second most recent images, in the size of the IPMN (that is, an increase from 8 mm to 12 mm) in the pancreatic head, while the dilation (6 mm) of the main pancreatic duct showed minimal change (axial image in Figure 1A and cholangiopancreatography in Figure 1B). The findings in the branch duct IPMN showed discrepant results with different imaging modalities. While contrast-enhanced computed tomography (Figure 1C) and magnetic resonance imaging did not show any nodules in the IPMN, endoscopic ultrasound revealed a suspected nodule (6 mm). The tumor markers carcinoembryonic antigen (CEA), carbohydrate antigen 19–9 (CA19-9), and DUPAN-2 were all within normal limits. Because there was an increase in the size of the branch duct IPMN, and a preoperative endoscopic ultrasound suggested the existence of a nodule in the IPMN, a malignancy in the branch duct IPMN was suspected and we performed pylorus-preserving pancreatoduodenectomy (PpPD) with lymphadenectomy for diagnostic and therapeutic purposes. The postoperative course was uneventful and the patient was discharged on post-operative Day 29 and has been alive for 18 months.

**Figure 2:**
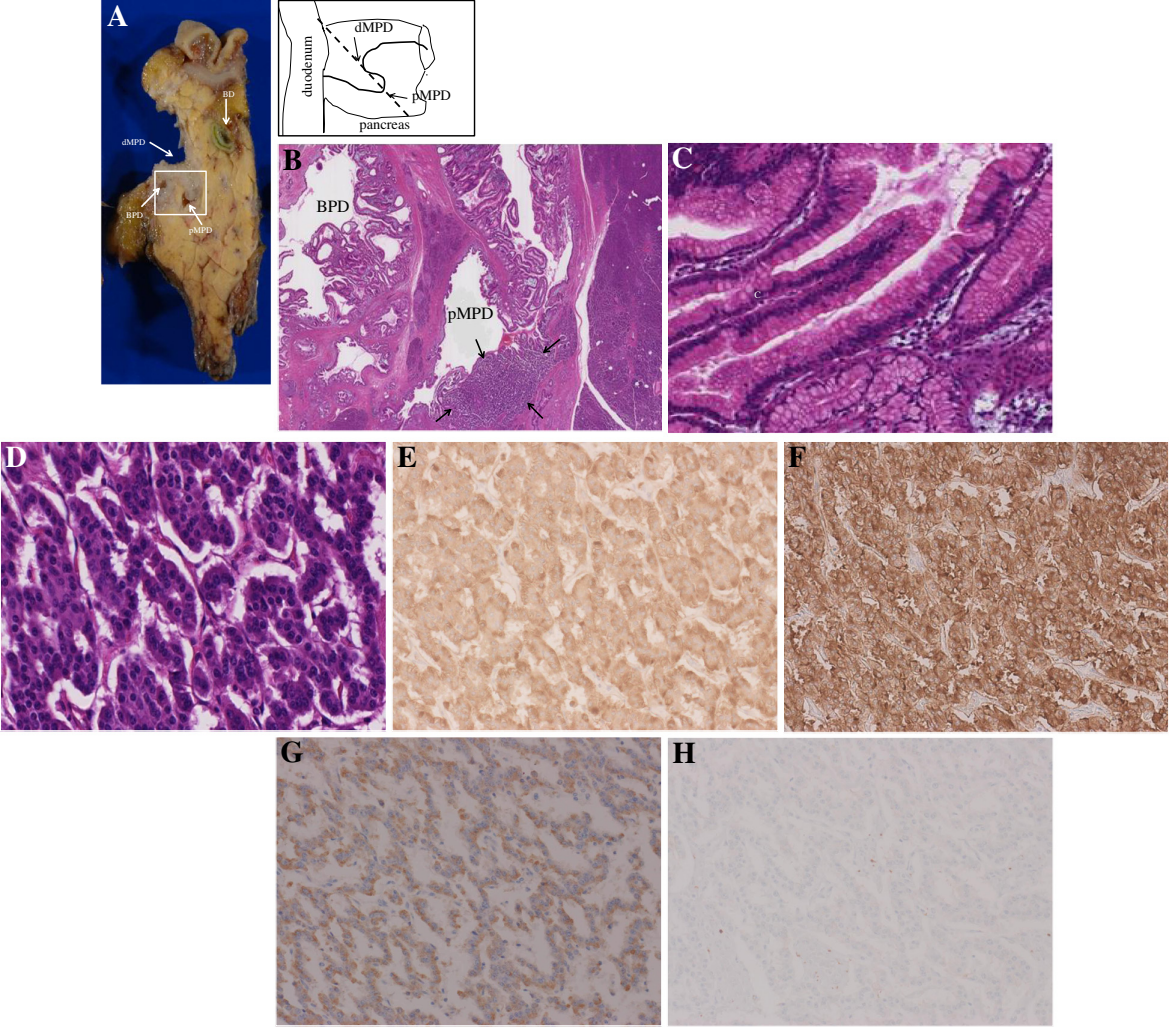
**Macroscopic and microscopic findings of the resected specimen. A**) Macroscopic findings of the resected specimen. The pancreas head was cut in the direction shown in the illustration. Because the main pancreas duct was markedly curved in the pancreas head, a proximal section (pMPD) and a distal section (dMPD) are seen in the same section. A cross section of the intra-pancreatic bile duct is seen (indicated as BD). In this section, one of the dilated branch pancreas ducts is seen (indicated as BPD). **B**) Microscopic findings of the resected specimen (hematoxylin and eosin stain, a loupe observation of the rectangle in macroscopic finding above). Almost all epithelia of both the main and branch pancreas ducts were composed of intraductal papillary mucinous adenoma with mild atypia. BPD and pMPD indicates dilated branch pancreas duct and proximal main pancreas duct, respectively. There is a demarcated area (surrounded by arrows) consisting of endocrine tumor. **C**) Microscopic findings of the intraductal papillary mucinous adenoma (x460 nm/pix). **D**) Microscopic findings of the neuroendocrine area (x460 nm/pix). **E-G**) Immunohistochemical examination of the endocrine cells was positive for chromogranin A (**E**), synaptophysin (**F**), and glucagon (**G**). **H**) Immunohistochemical examination of Ki-67. The positivity rate was less than 2%.

In the resected specimen, a dilated main pancreas duct, 6 mm in size, and a cluster of multiple dilated branch pancreas ducts were seen (Figure 2A). Histological examination revealed that both the main and branch pancreas ducts were composed of intraductal papillary mucinous adenoma with mild atypia (Figure 2B, C). Therefore, we diagnosed this patient as having mixed-type IPMN. Incidentally, a 3-mm nodule of endocrine cells was found in the IPMN lesion in the main pancreas duct (Figure 2B, D). Since the cells demonstrated positive staining for chromogranin A and synaptophysin (Figure 2E, F), the nodule was diagnosed as a PEN. The cells also showed positive staining for glucagon (Figure 2G), and negative staining for insulin and somatostatin. The plasma levels of glucagon, insulin and somatostatin had not been examined preoperatively. Since the mitotic count per 10 high-power fields was less than 2 and the Ki-67 index was less than 2% (Figure 2H), the PEN was pathologically classified as a well-differentiated neuroendocrine tumor and was low-grade (G1) according to the 2000 and 2010 WHO criteria, respectively [11,12]. We retrospectively assessed the preoperative images but did not find a nodule corresponding to the PEN lesion. Regarding the nodule in the branch duct IPMN, we also retrospectively assessed the preoperative images and resected specimen but did not find evidence of a nodule in the cluster of multiple dilated branch pancreas ducts corresponding to the endoscopic ultrasound finding. Thus, we recognize that such nodules can be misdiagnosed or over-diagnosed (that is, generate a false positive result) by endoscopic ultrasound.

## Discussion

Neuroendocrine tumors are neoplasms that arise from cells of the endocrine and nervous systems. They can originate within the pancreas and are quite distinct from the usual form of pancreatic cancer, which arises in the exocrine pancreas. IPMN is a type of neoplasm that grows within the pancreatic ducts and is characterized by the production of thick mucinous fluid. These two disease entities have been thought to be distinct and no tumorigenetic associations between them have been reported in the past. Therefore, the incidence of coexistence of these rare neoplasms should be extremely low if their individual incidences are simply multiplied. However, Marrache *et al.*[4] reported that the prevalence of association between these tumors was 2.8% at their institute (6 of 211 patients who underwent surgery for a PEN or IPMN). Goh *et al.*[5] and Gill *et al.*[10] subsequently reported prevalence rates of 4.6% (3 of 65 patients who underwent surgery for a PEN or IPMN) and 3.8% (4 of 104 patients who underwent surgery for IPMN), respectively. These studies suggest that occurrence of PEN and IPMN is more frequent than expected in the past and that potentially concomitant PEN and IPMN may be underdiagnosed. We retrospectively reviewed previous pathological reports of 34 PENs and 40 IPMNs in the past 15 years in our institute, but found no reports of concomitant PEN and IPMN in a total of 74 cases. Given the present case, the incidence of concomitant PEN and IPMN is 1.3% (1 of 75 patients who underwent surgery for PEN or IPMN) in our institute, which seems lower than the rates described above [3-5]. To determine the actual incidence of synchronous occurrence, it will be necessary to examine a large number of patients with PEN or IPMN and carefully examine resected specimens to determine the presence of concomitant PEN and IPMN.

Previous studies have discussed the tumorigenesis of concomitant PEN and IPMN. There are two major hypotheses: 1) one cell type in a unique tumoral process could transdifferentiate into another cell type; and 2) two cell types could arise from a common neoplastic progenitor. The former hypothesis is supported by many investigators. For example, Goh *et al.* noted that (i) the mean age of concomitant patients (60 years) corresponded approximately with that of IPMN, while PEN occurs in much younger patients, (ii) the dominant neoplasm was IPMN in most of the patients, and (iii) diagnosed PENs were of a non-functional nature in most of the patients, based on published reports, which suggests that the PEN component typically arises through transdifferentiation from IPMN [5]. Terada *et al.* found that argentaffin-, serotonin- and gastrin-secreting cells were present in IPMN but not in normal pancreatic ductal cells, and they suggested that IPMN has the potential for endocrine differentiation [13]. Hashimoto *et al.* found positivity for exocrine markers expressed on some endocrine tumor cells in a case of mixed PEN and IPMN, and it was noted that the endocrine tumor cells might transdifferentiate to ductal tumor cells [7]. All the features above may be suggestive of tumorigenesis, but is little better than speculation. The present case was a 74-year-old man, who had IPMN dominancy and showed positive glucagon staining in the PEN; however, this case did not provide definitive findings regarding tumorigenesis. We recognize that it is very difficult to assess the tumorigenesis of concomitant PEN or IPMN. It is important to not only assess patients for concomitant PEN and IPMN but also to describe the features with respect to tumorigenesis.

In a search of the PubMed database, we found 19 cases with concomitant PEN and IPMN in eight articles (Table 1). Together with our present case, there are 20 cases with concomitant PEN and IPMN (7 males and 13 females, mean age was 63.7 years old) described in the literature. The locations of PEN and IPMN were not described in nine cases, apparently distant in five cases (case no. 2, 3, 4, 5 and 7 in Table 1), and very close or mixed in the same tumor in six cases (case no. 1, 6, 11, 12, 13 and the present case in Table 1). The preoperative diagnosis was IPMN in 10 cases, PEN in 2 cases, not described in 2 cases, and concomitant PEN and IPMN in only 6 cases. In 6 of the 10 cases whose preoperative diagnosis was IPMN, the size of the PEN was less than 10 mm. The pathological features of PEN were benign in 12 cases, potentially malignant in 3 cases, neuroendocrine carcinoma in 4 cases, and not described in 1 case. The mean tumor sizes (maximum and minimum) of PEN depending on the pathological features were 9.2 (18, 2) mm in benign, 22.6 (28, 20) mm in potentially malignant, and 26.5 (35, 16) mm in neuroendocrine carcinoma. Based on these results from a review of the published literature, concomitant PEN and IPMN is frequently diagnosed as IPMN only and concomitant PEN goes undiagnosed due to its small size. The tumor size of PEN may serve as a guide for clinicopathological features irrespective of the existence of concomitant IPMN [14]. Although post-operative courses are not fully described in most cases, the prognoses for cases of neuroendocrine carcinoma seem pessimistic.

**Table 1:** Patients with concomitant pancreatic endocrine neoplasm and intraductal papillary mucinous neoplasm reported in the literature

**No. (ref.)**	**Age/Sex**	**Preoperative diagnosis**	**Surgery**	**PEN**		**IPMN**		**Postoperative outcome**
				**Location, size (mm)**	**Pathology1**	**Location, size (mm), type**	**Pathology2**	
1 (3)	51/M	PEN	DP	Tail, 15	Islet cell tumor with nesidioblastosis	Tail, ND, ND	IPMH (intraductal papillary mucinous hyperplasia)	ND
2 (4)	73/M	IPMN and PEN	DP	Tail, 28	Potentially malignant	Tail, ND, Branch	Benign	ND
3 (4)	40/F	IPMN and PEN	PD	Head, 11	Benign	Head, ND, Branch	Benign	ND
4 (4)	61/F	IPMN	DP	Tail, 12	Benign	Tail, ND, Mixed	Borderline	ND
5 (4)	55/F	PEN	PD	Head, 30	Malignant (duodenal wall invasion, peripancreatic lymph nodes metastases)	Head, ND, Mixed	Benign	ND
6 (4)	68/F	IPMN and PEN	DP	Body, 18	Benign	Body, ND, Mixed	Benign	ND
7 (4)	62/M	IPMN	PD	Head, 20	Potentially malignant	Head, ND, Mixed	Malignant noninvasive	ND
8 (5)	65/F	IPMN	TP	Body, 2	Benign	Head, Body, 40, Mixed	Malignant invasive	Disease-free, 10 months
9 (5)	66/M	IPMN	TP	Tail, 5	Benign	Entire, 150, Mixed	Malignant invasive	Alive, 70 months
10 (5)	58/M	IPMN	DP	Tail, 8	Benign	Tail, 18, Branch	Borderline	Alive, 5 months
11 (6)	72/F	ND	PD	Head, 25	PDNC (resional lymph nodes metastases)	Head, ND, ND	Borderline malignant potential	Died, 10 months
12 (7)	75/M	IPMN	PD	Head, 35	WDNC (peripancreatic lymph nodes metastases)	Head, 35, Mixed	Moderately to poorly differentiated adenocarcinoma	Died, 6 months
13 (8)	54/F	ND	PD	Head, ND	ND	Head, 25, Branch	Benign	ND
14 (9)	59/F	IPMN and PEN	observation	Body, 7.8	Benign	Body, Tail, 10 the largest, Branch	ND	Alive, 12 months
15 (9)	55/F	IPMN and PEN	enucleation	Head, 20	Low malignant potential	Head, 5,6,7, Branch	ND	ND
16 (10)	67/M	IPMN	TP	Head, 8	WDNT	Diffuse, 20, Main	Low grade dysplasia	ND
17 (10)	72/F	IPMN and PEN	DP	Tail, 16	WDNC (peripancreatic lymph nodes metastases)	Body, 9, Branch	Low grade dysplasia	ND
18 (10)	72/F	IPMN	DP	Body, 9	WDNT	Body, 15, Branch	Low grade dysplasia	ND
19 (10)	76/F	IPMN	TP	Head, 11	WDNT	Head, 27, Branch	Well differentiated adenocarcinoma	ND
20	74/M	IPMN	PD	Head, 3	WDNT/NET G1	Head, 10, Mixed	Low grade dysplasia	Alive, 12 months

1. Pathology of PEN is described according to WHO 2000 criteria, as this was used in all prior studies. As for the present case, the pathology of the PEN is described according to both WHO 2000 and 2010 criteria.

2. The pathology of IPMN is described according to criteria in prior studies [15]. As for the present case, the pathology of IPMN is described according to WHO 2010 criteria [16].

Branch, branch type IPMN; DP, distal pancreatectomy; G1, Neuroendocrine tumor G1; IPMN, intraductal papillary mucinous neoplasm; Main, main duct type IPMN; Mixed, mixed type IPMN; ND, not described; PD, pancreatoduodenectomy; PDNC, poorly differentiated neuroendocrine carcinoma; PEN, pancreatic endocrine neoplasm; TP, total pancreatectomy; WDNC*,* well differentiated neuroendocrine carcinoma; WDNT, well-differentiated neuroendocrine tumor.

It is important to determine whether the postoperative course and ideal management of cases of concomitant PEN and IPMN differ from that of cases of PEN only or IPMN only. No definitive guidelines have been established in previous case reports of concomitant PEN and IPMN due to the small number of reported cases. Since there have been no reports of cases with concomitant PEN and IPMN in which both PEN and IPMN showed benign or low-grade malignancy, but one or the other showed oncologically aggressive behavior, we believe that there are no synergistic effects between PEN and IPMN, and that cases of concomitant PEN and IPMN should be managed according to their respective natural histories. At the present time, this suggestion is speculative and needs to be validated based on a larger number of cases in the future.

## Conclusion

In conclusion, we encountered a case of concomitant PEN and IPMN. There have been some reports suggesting that the occurrence of concomitant PEN and IPMN has been underreported or undetected because of lack of awareness of the potential for concomitance and poor examination of specimens. It is important to recognize the concomitant neoplasms and determine the actual incidence of this occurrence. We hope that this case presentation will serve as a stimulus for further studies to identify concomitant PEN/IPMN and to better understand the underlying mechanisms.

## Consent

Written informed consent was obtained from the patient for publication of the Case report and accompanying images. A copy of the written consent is available for review by the Editor-in-Chief of this journal.

## Abbreviations

BPD: Branch pancreas ducts; CA19-9: Carbohydrate antigen 19–9; CEA: Carcinoembryonic antigen; dMPD: Distal section of the main pancreas duct; IPMN: Intraductal papillary mucinous neoplasm; MPD: Main pancreas duct; PEN: Pancreatic endocrine neoplasm; pMPD: Proximal section of the main pancreas duct; PpPD: Pylorus-preserving pancreatoduodenectomy; WHO: World Health Organization.

## Competing interests

The authors declare that they have no competing interests.

## Author’s contributions

YK wrote the paper. MS participated in the operation and supervised the writing of the paper. MT conducted the operation and supervised the writing of the paper. YM, HT and MSakamaoto prepared the pathological findings. AU and AT prepared the radiological images. GO and RN participated in the writing of the paper. MTanaka and KM participated in the operation. MK, OI, SK and KA supervised the writing of the paper. YKitagawa represents our surgical department and supervised the writing of the paper. All authors read and approved the final manuscript.
